# Quantitative classification of melasma with photoacoustic microscopy: a pilot study

**DOI:** 10.1117/1.JBO.29.S1.S11504

**Published:** 2023-11-03

**Authors:** Zhiyang Wang, Yuying Chen, Shu Pan, Wuyu Zhang, Ziwei Guo, Yuzhi Wang, Sihua Yang

**Affiliations:** a South China Normal University, Institute of Laser Life Science, College of Biophotonics, MOE Key Laboratory of Laser Life Science, Guangzhou, China; b South China Normal University, College of Biophotonics, Guangdong Provincial Key Laboratory of Laser Life Science, Guangzhou, China; c Guangdong Photoacoustic Technology Co., Ltd., Foshan, China; d Zhujiang Hospital of Southern Medical University, Department of Plastic Surgery, Guangzhou, China; e General Hospital of Southern Theater Command, Department of Burns and Plastic Surgery, Guangzhou, China

**Keywords:** medical dermatology, photoacoustic microscopy, melasma, imaging technology, quantitative classification

## Abstract

**Significance:**

The classification of melasma is critical for correct clinical diagnosis, treatment selection, and postoperative measures. However, preoperative quantitative determination of melasma type remains challenging using conventional Wood’s lamp and optical dermoscopy techniques.

**Aim:**

Using photoacoustic microscopy (PAM) to simultaneously obtain the two diagnostic indicators of melanin and blood vessels for melasma classification and perform quantitative analysis to finally achieve accurate classification, rather than relying solely on physicians’ experience.

**Approach:**

First, the patients were classified by experienced dermatologists with Wood’s lamp and optical dermoscopy. Next, the patients were examined *in vivo* using the PAM imaging system. Further, the horizontal section images (X-Y plane) of epidermal melanin and dermal vascular involvement were extracted from the 3D photoacoustic imaging results, which are important basis for PAM to quantitatively classify melasma.

**Results:**

PAM can quantitatively reveal epidermal thickness and dermal vascular morphology in each case and obtain the quantitative diagnostic indicators of melanin and blood vessels. The mean vascular diameter in lesional skin (223.2  μm) of epidermal M+V-type was much larger than that in non-lesional skin (131.6  μm), and the mean vascular density in lesional skin was more than three times that in non-lesional skin. Importantly, vascular diameter and density are important parameters for distinguishing M type from M+V type.

**Conclusions:**

PAM can obtain the data of epidermal thickness, pigment depth, subcutaneous vascular diameter, and vascular density, and realize the dual standard quantitative melasma classification by combining the parameters of melanin and blood vessels. In addition, PAM can provide new diagnostic information for uncertain melasma types and further refine the typing.

## 1 Introduction

Melasma, a chronic, recurrent pigmented skin disorder predominantly affecting women’s faces, stems from various factors.^1^ Its accurate classification is pivotal for developing efficient treatments and guiding future therapy strategies.^2^ However, melasma presents diverse complex features, and clinical diagnosis includes multiple classification criteria based on melasma location, the depth distribution of melanin in skin, and involvement of subcutaneous blood vessels. Current available diagnostic tools for melasma include Wood’s lamp, confocal microscopy, and optical dermoscopy.^3^ Wood’s lamp examination is the most widely used method of melasma classification by highlighting the difference in pigmentation of the lesioned skin.^4^ Confocal microscopy can detect melanin and melanocytes *in vivo* with high resolution, and its imaging results are closely related to histopathologic findings.^5^ Another important technique, optical dermoscopy allows a non-invasive method to examine the skin, which facilitates the visualization of pigment components and their location on the skin layers.^6^ However, using the above techniques remains a challenge to achieve accurate melasma classification, for example, there are cases diagnosed as epidermal melasma using Wood’s lamp and optical dermoscopy, but confocal imaging can still reveal pigmentation in both the epidermis and dermis. Also, these devices yield single-faceted results and fail to simultaneously provide scientific and objective parameters for melanin and vascular involvement at the lesion site. Further classification often necessitates subjective visual evaluation by experienced dermatologists. Skin biopsy remains the gold standard for melasma classification but is used with caution due to its invasive and cumbersome nature. Current melasma classification requires newer non-invasive diagnostic tools, especially an imaging device capable of simultaneously quantifying subcutaneous melanin and blood vessels, which can offer novel insights for choosing the most effective treatment, evaluating therapeutic outcomes, and reclassifying ambiguous melasma.^7^

Emerging high-resolution optical imaging techniques, such as optical coherence tomography (OCT), two-photon microscopy, and optical polarization imaging, can provide new insights into the clinical diagnosis of dermatosis.^8^^–^^11^ In general, pure optical imaging modalities are difficult to simultaneously visualize melanin and blood vessels with high contrast, and the imaging resolution of deep tissue layers is affected by strong photon scattering. As a hybrid imaging technique, photoacoustic imaging (PAI) has shown great potential in clinical (preclinical) diagnosis.^12^^–^^19^ In particular, photoacoustic microscopy (PAM) imaging modality enables label-free high-resolution quantitative imaging of the skin through high optical absorption in endogenous hemoglobin and melanin^20^^–^^27^ and simultaneously reconstructs the three-dimensional (3D) morphological structure of melanin and blood vessels.^28^^–^^30^ At present, PAM has been successfully applied in the clinical (preclinical) diagnosis of psoriasis,^31^^,^^32^ port wine stain,^33^^,^^34^ melanoma,^35^^,^^36^ and other human skin diseases.^37^^–^^39^ However, the imaging potential of PAM for melasma has not yet been demonstrated, especially in research on quantitative classification of lesion grading. Given the clinical potential of PAM, it is expected to enhance the current clinical diagnosis and treatment of melasma and solve the problem that conventional techniques cannot quantitatively distinguish multiple endogenous pigments in the skin. In this study, PAM technology was performed to obtain the key 3D morphological structures of melanin and blood vessels in melasma and quantitatively analyze their density and depth in the epidermis and dermis of the skin. This strategy integrates photoacoustic data of melanin and blood vessels to quantitatively classify melasma, rather than relying solely on physicians’ experience. Before the imaging experiments, the sites diagnosed by experienced dermatologists to have melasma are defined as lesional skin, where melanin increases in the epidermal layer of skin and may infiltrate into the dermis. The non-lesional skin (normal skin) refers to the sites without abnormal pigment accumulation and is used as control sites. Four melasma volunteers were selected to be classified with the proposed method, and the PAI results were compared with clinical diagnosis. It was found that this method can accurately and effectively classify melasma and can provide more abundant and accurate quantitative information than the existing equipment. To our knowledge, this is the first time that PAM has been introduced to classify melasma. This method overcomes the limitations of conventional detection equipment and opens up a new way to achieve accurate and refined melasma classification.

## 2 Materials and Methods

### 2.1 PAM System

**Fig. 1:**
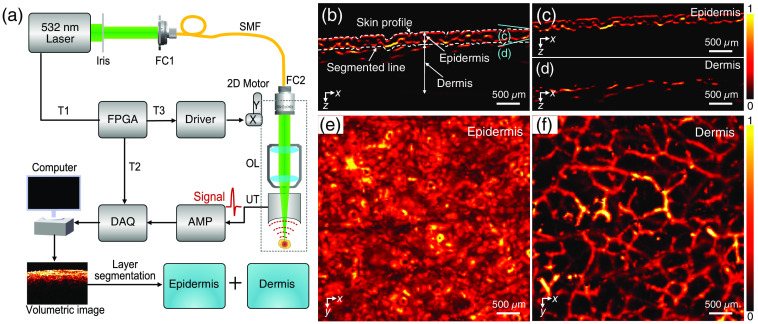
Schematic diagram of the PAM system and skin layer segmentation. (a) Schematic diagram of the PAM system; FC1, fiber coupler; FC2, fiber collimator; SMF, single-mode fiber; OL, objective lens; UT, ultrasonic transducer; T1, T2, T3, trigger; DAQ, data acquisition; FPGA, field programmable gate array; AMP, amplifier. (b) A single B-scan PA image. (c) Segmented epidermal layer from (b). (d) Segmented dermal layer from (b). (e) PA lateral (X-Y plane) MAP images of epidermal layer. (f) PA lateral (X-Y plane) MAP images of dermal layer.

A commercially available PAM equipment (PASONO-SKIN, Guangdong Photoacoustic Technology Co., Ltd., Foshan, China) was utilized in this study. The schematic diagram of the PAM system is shown in Fig. 1(a). The core integrated imaging probe is composed of single-mode fiber, fiber collimator, objective lens, high-frequency ringed ultrasonic transducer (central frequency: 35 MHz, bandwidth: 15 to 55 MHz, focal length: 8 mm), and coupling cup. When performing human skin imaging, the coupling cup needs to touch the skin with deionized water, and the two-dimensional (2D) scanning platform drives the integrated imaging probe to perform raster scanning at a speed of 10 mm/s. Considering that both melanin and hemoglobin have strong absorption at 532-nm wavelength, a 532-nm nanosecond pulsed laser was used as the excitation source of PA signals. A low-noise preamplifier (LNA-650, RFBAY) is used to amplify the photoacoustic signal, and then the amplified signal passes through a bandpass filter. Each laser pulse generated an A-line signal in the imaging depth direction, and B-scan tomography image composed of continuous A-line signals was obtained by 2D transverse scanning. For the spatial resolution of the imaging system, our previous experiments showed that the PAM system has a lateral resolution of about 7  μm and an axial resolution of about 50  μm.^28^^,^^33^

### 2.2 Data Processing and Statistical Analysis

For 3D photoacoustic skin image reconstruction, each A-line signal is processed by median and filtering, then linearly projected to obtain a B-Scan image, and all B-Scan images are synthesized to display the 3D skin tissue structure. Normal 3D skin includes epidermis (stratum corneum and stratum basale), dermis, and subcutaneous tissue. These skin layers are distributed at different depths and have different morphological structures, which can be distinguished by depth information.^28^^,^^32^^,^^40^ In the quantitative melasma classification, to accurately distinguish the distribution characteristics of melanin and blood vessels in different skin layers, a skin tissue stratification algorithm based on MATLAB (R2019b, MathWorks) was programmed to accurately segment skin tissue at different depths. The specific implementation steps were as follows: first, a threshold value is set to reduce the influence of the background noise in the imaging system. Then, the first extreme point of each A-line signal is extracted one by one to form the skin profile line [Fig. 1(b)]. A certain depth from the skin profile is defined as the segmented line in B-scan image, which is attempted based on different epidermal layer thicknesses. Finally, the PA data are intercepted according to the segmented line to obtain photoacoustic images of different skin layers, such as melanin in the epidermis [Figs. 1(c) and 1(e)] and blood vessels in the dermis [Figs. 1(d) and 1(f)]. Moreover, the parameters of melanin and blood vessels can be quantified based on the reconstructed 3D photoacoustic images. We converted the imaging results into grayscale images to analyze melanin in lesional and non-lesional skin. The mean diameter and density of blood vessels were analyzed by counting the vascular pixel values, where the vascular density was defined as the ratio of all vascular pixels in the given area to the total area pixels.

### 2.3 Classification Method with PAM

**Fig. 2:**
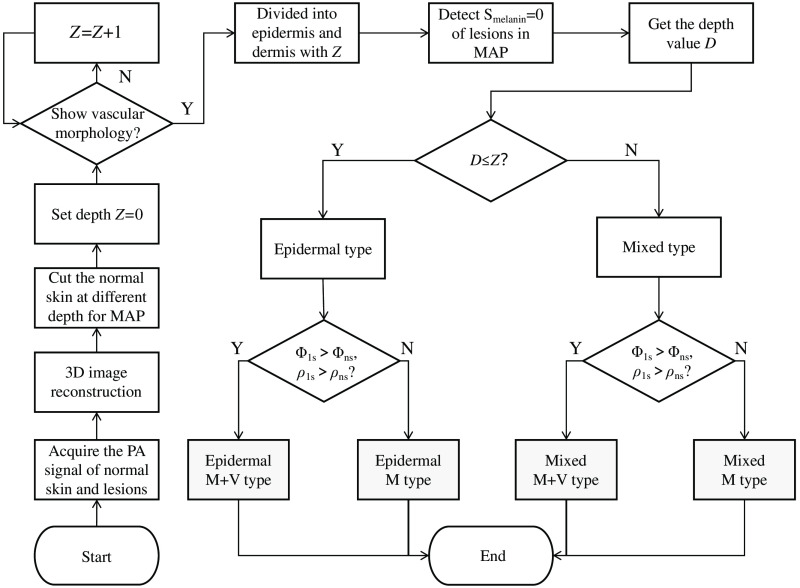
Flow chart of melasma classification with PAM. 3D, three-dimensional; MAP, maximum amplitude projection; Z, selected sampling depth (0-Z) in Aline data, and the final determined Z is the epidermal thickness. Y, yes; N, no; D, the depth when the area of melanin is 0; $\Phi_{\mathrm{ls}}$, the average diameter of blood vessels in the lesional skin; $\Phi_{\mathrm{ns}}$, the average diameter of blood vessels in the non-lesional skin; $\rho_{\mathrm{ls}}$, the average density of blood vessels in the lesional skin; $\rho_{\mathrm{ns}}$, the average density of blood vessels in the non-lesional skin; M + V type, melanized with vascularized type; M type, melanized type.

The current classification of melasma is complex and overlapping, for example, some epidermal and mixed types of melasma are not only simply pigmentary abnormalities but also accompanied by vascular involvement. In this case, the imaging depth and specificity of PAM have advantages over conventional optical dermoscopy and confocal microscopy. With the characteristics of PAM, we first located the cortex where melanin was located, then analyzed whether its formation was related to blood vessels, finally subdivided melasma into epidermal M type, epidermal M+V type, mixed M type, and mixed M+V type. The steps of the proposed photoacoustic classification strategy are shown in Fig. 2. The imaging probe of PAM was used to successively aim at non-lesional skin and lesional skin of each patient. Since the skin thickness of each patient is different, each volunteer’s epidermis and dermis thickness needs to be identified from the 3D photoacoustic images. First, the skin layering algorithm was used to extract the first extreme point of each A-line signal in the patient’s B-scan image to form the skin contour. Second, set the skin contour line as the reference coordinate, and select a certain depth downward until the morphological features of blood vessels are found on the cross-sectional maximum amplitude projection (MAP) image. Finally, the depth Z is defined as the dividing line between the epidermis and the dermis. After identifying the thickness of different skin layer, the next step was to locate the depth of pigmentation in lesional skin. If the pigment was distributed only in epidermis, it was epidermal type. If the pigment was distributed in both epidermis and dermis, it was mixed type. Finally, we analyzed the vascular morphology of dermis and compared the mean diameter and mean density of vessels in lesional skin and non-lesional skin. If the mean diameter and mean density of vessels in the lesional skin were greater than those in non-lesional skin, it was M+V type, otherwise were M type. Eventually, melasma was reclassified into epidermal M type, epidermal M+V type, mixed M type, and mixed M+V type.

## 3 Results

### 3.1 Verify the Classification by PAM

**Fig. 3:**
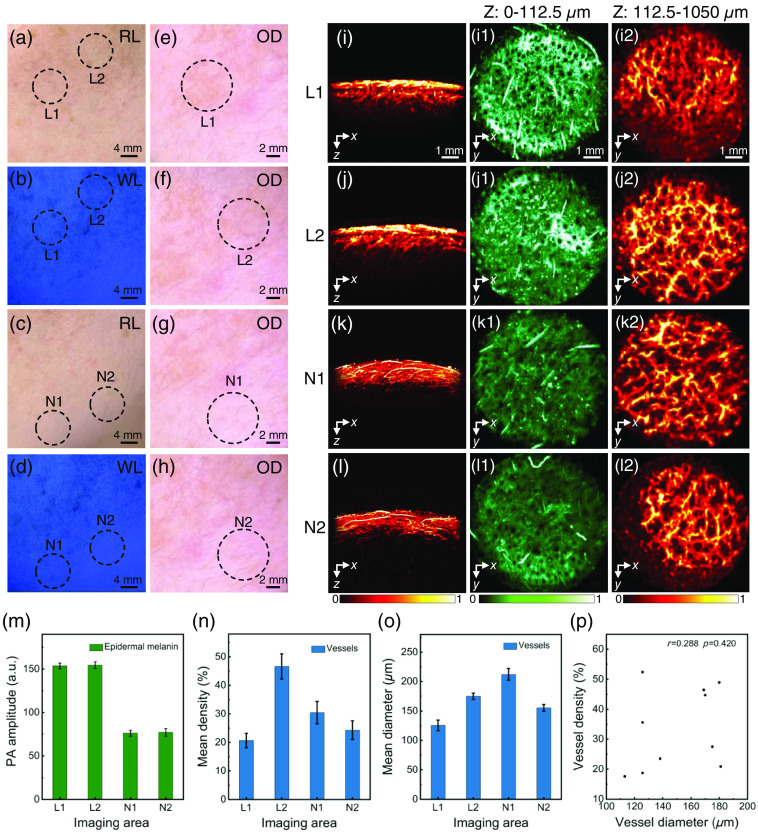
Images and statistics of lesions and normal areas of epidermal M type (patient 1) under room light, wood’s lamp, optical dermoscopy, and PAM. (a) Lesions under room light; (b) lesions under wood’s lamp; (c) normal areas under room light; (d) normal areas under Wood’s light; (e), (f) lesions under optical dermoscopy; (g), (h) normal areas under optical dermoscopy; (i)–(l2) photoacoustic images; (m) PA amplitude of epidermal melanin; (n) mean diameter of dermal vessels; (o) mean density of dermal vessels; and (p) correlation statistics of the diameter and density of vessels in the lesions. L1, L2, imaging area of lesional skin; N1, N2, imaging area of non-lesional skin; RL, room light; WL, Wood’s lamp; OD, optical dermoscopy.

First, before the human skin was imaged, the volunteers were first required to remove makeup and clean the entire face to rule out the influence of cosmetics on the imaging results. Experienced dermatologists classified the volunteers using conventional detection tools and then performed PAI. Imaging results of epidermal M type were shown in Fig. 3 (patient 1). The color contrast between lesional skin and non-lesional skin under Wood’s lamp was enhanced, and light brown reticular patches without vascular structure were visualized under the optical dermoscopy [Figs. 3(a)–3(h)], so the dermatologist diagnosed it as epidermal M type melasma. The X-Z cross-sectional MAP images were shown in Figs. 3(i)–3(l), demonstrating that the melanin in the lesion sites showed strong optical absorption properties in the epidermis. The X-Z cross-sectional MAP images were shown in Figs. 3(i1)–3(l2), indicating the absorption of laser by melanin and blood vessels. According to the defined boundary between the epidermis and dermis, for this volunteer, the subcutaneous 0∼ to 112.5  μm is the epidermis, and the depth below 112.5  μm is the dermis. It was also found that there was no significant difference and correlated relationship in the vascularity [Figs. 3(m)–3(p), r=0.288, P>0.05]. Based on above analysis, the type of melasma was epidermal M type, with melanin deposition in the subcutaneous depth of 0 to 112.5  μm.

**Fig. 4:**
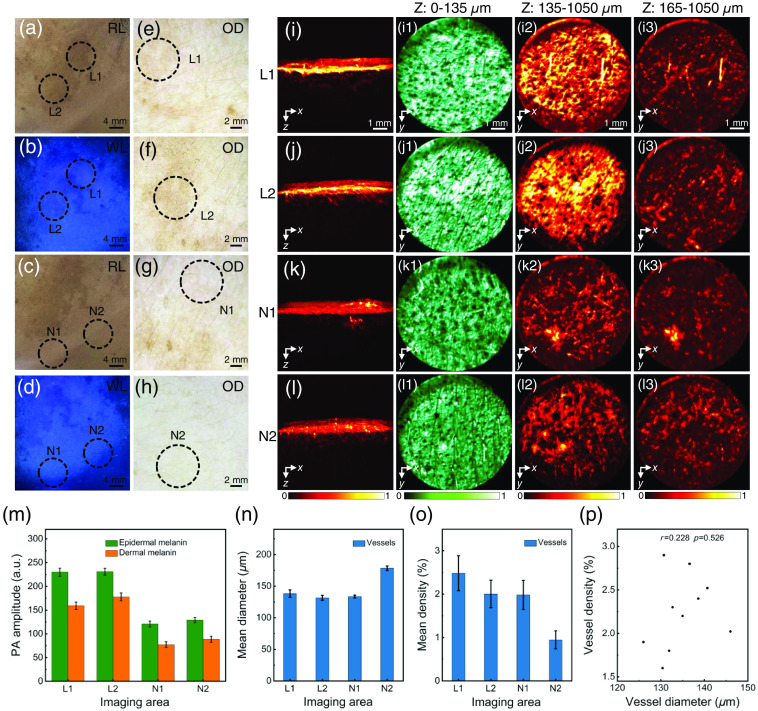
Images and statistics of lesions and normal areas of mixed M type (patient 2) under room light, Wood’s lamp, optical dermoscopy, and PAM. (a) Lesions under room light; (b) lesions under Wood’s lamp; (c) normal areas under room light; (d) normal areas under Wood’s light; (e), (f) lesions under optical dermoscopy; (g), (h) normal areas under optical dermoscopy; (i)–(l3) photoacoustic image; (m) PA amplitude of melanin; (n) mean diameter of dermal vessels; (o) mean density of dermal vessels; and (p) correlation statistics of the diameter and density of vessels in the lesions.

Imaging results of mixed M type was shown in Fig. 4 (patient 2). Differences in pigmentation of lesional and non-lesional skin under Wood’s lamp are highlighted, and more melanin accumulated at the lesional sites [Figs. 4(a)–4(d)]. As with the epidermal M type, dark brown reticular plaques were visible under optical dermoscopy [Figs. 4(e)–4(h)], but no vascular structures were revealed. This volunteer was diagnosed mixed M type by experienced dermatologists. According to the structural characteristics of the 3D photoacoustic images, it can be obtained that the thickness of the epidermis is 135  μm, and the dermis is below 135  μm of the skin surface. Similarly, the PA signal in lesional skin was stronger than that in non-lesional skin [Fig. 4(m)]. Dense punctate melanin was shown in the lesional skin, which was distributed in the interstitial spaces of the blood vessels [Figs. 4(i2)–4(j2)]. Through fine segmentation, it can be analyzed that melanin deposits at the lesional skin to a depth of 165  μm below the skin surface [Figs. 4(i3)–4(j3)], reaching the dermis. Another factor, the morphology of blood vessels between lesioned skin and non-lesional skin had no significant difference and correlation [Figs. 4(n)–4(p), r=0.228, P>0.05]. Based on above analysis, the volunteer was diagnosed as mixed M type melasma, with melanin deposited at a depth of 0 to 165  μm.

**Fig. 5:**
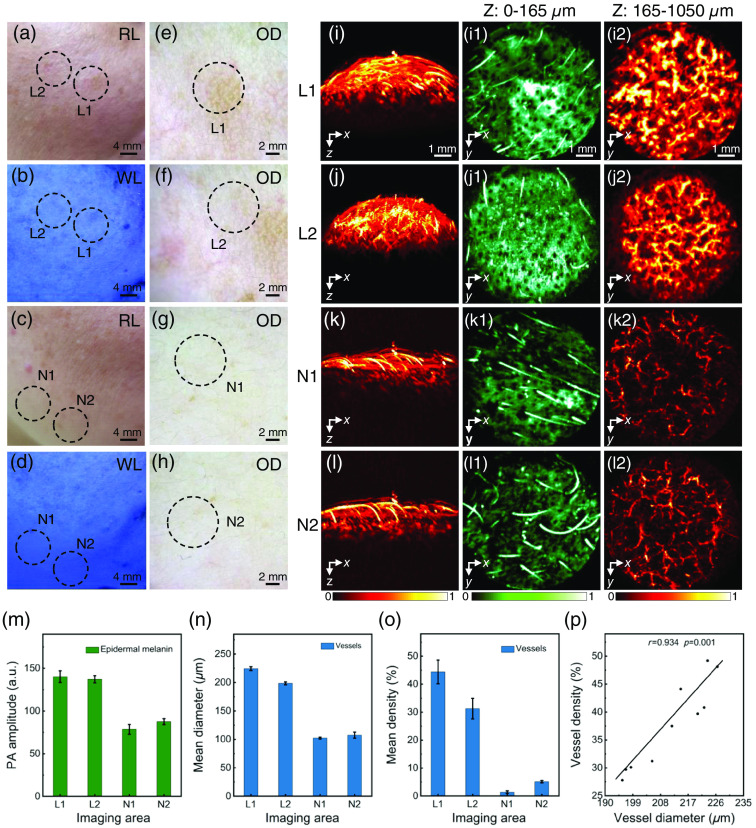
Images and statistics of lesions and normal areas of epidermal M+V type (patient 3) under room light, Wood’s lamp, optical dermoscopy, and PAM. (a) Lesions under room light; (b) lesions under Wood’s lamp; (c) normal areas under room light; (d) normal areas under Wood’s light; (e), (f) lesions under optical dermoscopy; (g), (h) normal areas under optical dermoscopy; (i)–(l2) photoacoustic image; (m) PA amplitude of epidermal melanin; (n) mean diameter of dermal vessels; (o) mean density of dermal vessels; and (p) correlation statistics of the diameter and density of vessels in the lesions.

Imaging results of epidermal M+V type was shown in Fig. 5 (patient 3). The pigmentation of lesional skin under Wood’s lamp is more obvious than that of non-lesional skin [Figs. 5(a)–5(d)]. Brown reticular plaques and reddish capillaries can be detected under optical dermoscopy [Figs. 5(e)–5(h)], and this volunteer was diagnosed as epidermal M+V type. According to the PA results, the depth of 0 to 165  μm under the skin surface is the epidermis, and the depth below 165  μm is the dermis. Further morphological observation found that melanin was only distributed in the epidermis. Figures 5(i2)–5(l2) provided the vascular morphology of the dermis, showing that blood vessels in lesioned skin are larger and denser than those in non-lesions skin. The statistical results further demonstrated that the PA signal, mean vascular diameter, and density were significantly stronger in lesional skin [Figs. 5(m)–5(o)]. Importantly, vascular diameter and density in lesional skin were statistically significant and strongly correlated [Fig. 5(p), r=0.934, P<0.05]. Based on the photoacoustic analysis results, the volunteer was diagnosed as epidermal M+V type melasma, and melanin was only deposited in the epidermal layer at a depth of 0 to 165  μm.

### 3.2 Reclassification of Indeterminate Melasma by PAM

**Fig. 6:**
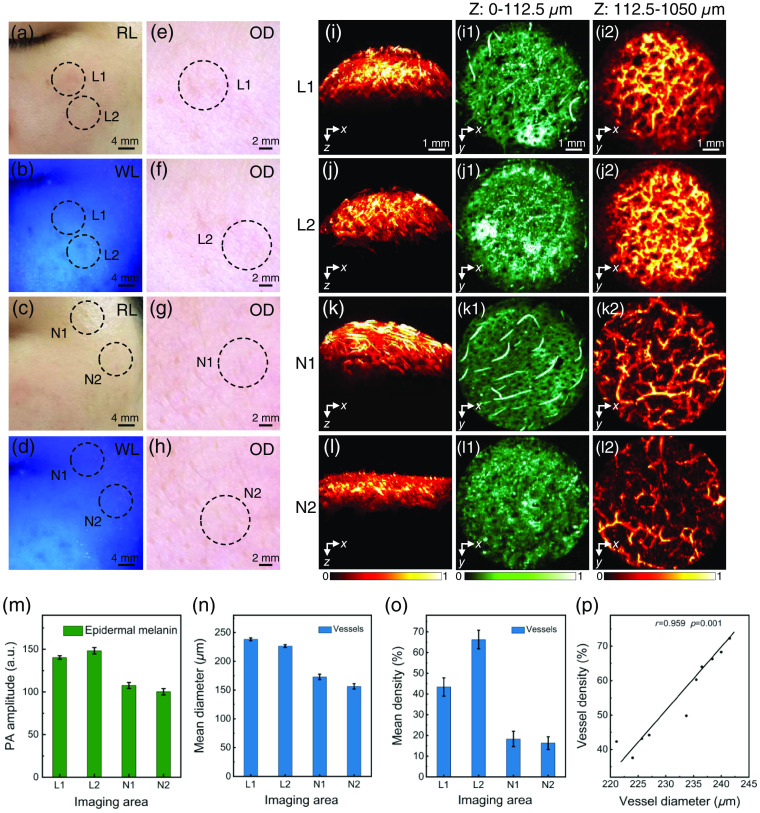
Images and statistics of lesions and normal areas of indeterminate type (patient 4) under room light, Wood’s lamp, optical dermoscopy, and PAM. (a) Lesions under room light; (b) lesions under Wood’s lamp; (c) normal areas under room light; (d) normal areas under Wood’s light; (e), (f) lesions under optical dermoscopy; (g), (h) normal areas under optical dermoscopy; (i)–(l2) photoacoustic image; (m) PA amplitude of epidermal melanin; (n) mean diameter of dermal vessels; (o) mean density of dermal vessels; and (p) correlation statistics of the diameter and density of vessels in the lesions.

There are some clinical cases of melasma that are difficult to determine its type by conventional Wood’s lamp and optical dermoscopy [Figs. 6(a)–6(h)], and dermatologists generally classify these cases as indeterminate type. Figure 6 provides the imaging results of indeterminate melasma (patient 4). First, the 3D PA images were segmented to obtain the epidermis with a depth of 0 to 112.5  μm [Figs. 6(i1)–6(l1)] and the dermis below 112.5  μm in depth [Figs. 6(i2)–6(l2)]. By observing the structural characteristics of the dermis, there is no accumulation of melanin in the interstitial space of blood vessels. Figures 6(m)–6(o) show that the PA signal, mean vascular diameter, and density in lesional skin are significantly stronger than those in non-lesional skin, and the vascular diameter and density in lesional skin are statistically significant and strongly correlated [Fig. 6(p), r=0.959, P<0.05]. Based on the above analysis, it can be concluded that the indeterminate melasma was epidermal M+V type.

## 4 Discussion

A total of four patients (age 27 to 48 years, Asian women) with melasma on the cheek were recruited for this study, and two patients were Fitzpatrick skin types III and two were skin types IV. Since there were few clinical cases of mixed M+V type, the enrolled patients in this study were epidermal M type, epidermal M+V type, mixed M type, and indeterminate type. For each patient, two lesional skin and two non-lesional skin were selected for PAI, and then the epidermis and dermis were segmented and analyzed. The pigmentation depth, mean vascular diameter, and vascular density measured by the PAM imaging system are shown in Table 1. Both epidermal type and mixed type had no vascular involvement. For epidermal M+V melasma, the mean vascular diameter and density in the dermis of the lesional skin are significantly higher than those of the non-lesional skin.

**Table 1:** Pathology information and PA results of melasma patients.

Case	Clinical classification	PA classification	Depth of pigmentation (μm)	Mean diameter (μm)		Mean density (%)	
				LS	NS	LS	NS
P1	Epidermal M	Epidermal M	112.5	150.3	163.8	38.6	26.4
P2	Mixed M	Mixed M	165.0	144.9	156.0	2.3	1.5
P3	Epidermal M+V	Epidermal M+V	165.0	214.1	104.5	40.7	4.3
P4	Indeterminate	Epidermal M+V	112.5	232.3	158.7	54.8	18.5

**Fig. 7:**
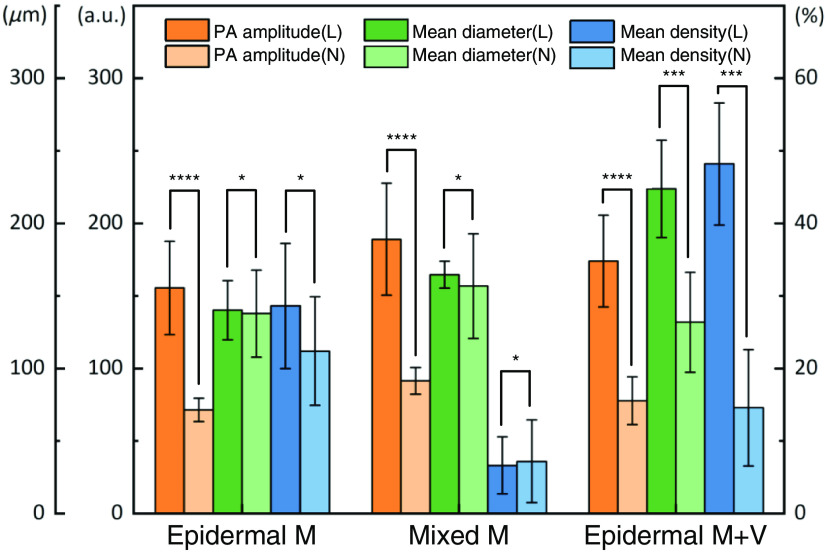
Statistics of photoacoustic amplitude, mean vascular density, and mean vascular diameter parameters in four enrolled patients with melasma. PA, photoacoustic; L, lesional skin; N, non-lesional skin. *P<0.05; ***P<0.001; ****P<0.0001.

Figure 7 shows the statistics of photoacoustic data, and the results show that mean vascular diameter and vascular density were the important parameter for distinguishing M type and M+V type. The mean vascular diameter in lesional skin of epidermal M+V type (223.2  μm) was much larger than that in non-lesional skin (131.6  μm), and the mean vascular density in lesional skin was more than three times that in non-lesional skin. P value of mean vascular diameter and mean vascular density in epidermal M+V type was more significant than that in epidermal M type and mixed M type. This study demonstrates that PAM can be used to provide quantitative parameters for melasma classification.

In this study, facing the current challenges in clinical diagnosis and classification of melasma, PAM was proposed to classify melasma. Four different types of melasma cases were imaged, demonstrating the potential and value of PAI technology in melasma classification. Future studies need to include more subjects to verify the universal application of this imaging technology. In addition, to better expand the application of PAI technology, multispectral PAM is expected to be applied to melasma classification, which can quantitatively resolve melanin and hemoglobin through spectral analysis rather than depth segmentation. For quantitative analysis of 3D PA data, it is hoped that a more accurate statistical method can be developed for vascular parameters of 3D region in future studies.

## 5 Conclusions

In summary, PAM provides a quantitative and non-invasive imaging technology to classify melasma. Although larger-scale studies are required to validate the technique’s capability and feasibility, we believe that the results of our initial pilot study show great potential for PAM as an accurate tool to quantify the melanin and subcutaneous vessels of melasma skin. First, we determine the epidermal thickness to segment the 3D photoacoustic data, then obtained morphological images of the epidermis and dermis, and finally analyzed the distribution depth of melanin and the morphological parameters of blood vessels. Photoacoustic analysis results are consistent with the judgment of experienced dermatologists. It is worth noting that for clinically indeterminate type (patient 4), PAI was reclassified into epidermal M+V type based on melanin and vascular involvement, which provides valuable information for further clinical treatment. In future clinical studies, PAM can monitor the recovery state of melanin and blood vessels in the lesion sites during the treatment of melasma, which is expected to dynamically adjust the treatment strategy.

## Data Availability

Data and code developed in this study are available upon reasonable request to the corresponding author.
